# The “I” in us, or the eye on us? Regulatory focus, commitment and derogation of an attractive alternative person

**DOI:** 10.1371/journal.pone.0174350

**Published:** 2017-03-20

**Authors:** David Rodrigues, Diniz Lopes, Madoka Kumashiro

**Affiliations:** 1 Instituto Universitário de Lisboa (ISCTE-IUL), CIS-IUL, Lisboa, Portugal; 2 Goldsmiths, University of London, London, United Kingdom; Technion Israel Institute of Technology, ISRAEL

## Abstract

When individuals are highly committed to their romantic relationship, they are more likely to engage in pro-relationship maintenance mechanisms. The present research expanded on the notion that commitment redirects self-oriented goals to consider broader relational goals and examined whether commitment interacts with a promotion and prevention focus to activate derogation of attractive alternatives. Three studies used cross-sectional and experimental approaches. Study 1 showed that romantically involved individuals predominantly focused on promotion, but not prevention, reported less initial attraction to an attractive target than single individuals, especially when highly committed to their relationship. Study 2 showed that romantically involved individuals induced in a promotion focus, compared to those in prevention focus, reported less initial attraction, but only when more committed to their relationship. Regardless of regulatory focus manipulation, more committed individuals were also less likely to perceive quality among alternative scenarios and to be attentive to alternative others in general. Finally, Study 3 showed that romantically involved individuals induced in promotion focus and primed with high commitment reported less initial attraction, than those primed with low commitment, or than those induced in prevention focus. Once again, for these latter no differences occurred according to commitment prime. Together, the findings suggest that highly committed promotion focused individuals consider broader relationship goals and activate relationship maintenance behaviors such as derogation of attractive alternatives to promote their relationship.

## Introduction

Why are some individuals in romantic relationships better at promoting relationship growth by warding off potential partners, whereas others find it more difficult abstaining from actively pursuing other relational interests? Maintaining a romantic relationship is not an easy task. Individuals must often forego pure self-motivations to engage in pro-relationship maintenance mechanisms. Regulatory Focus Theory [1] offers a possible framework for understanding which goals are activated under these conflicting situations. Promotion and advancement is associated with motivations to attain positive outcomes (e.g., gaining potential partners or being in a healthy relationship), whereas prevention is associated with avoiding negative outcomes (e.g., losing a partner or experiencing conflicts). Promotion, in particular, directs individuals to actively pursue desired goals, but for romantically involved individuals, desired goals may present a dilemma of choosing between pursuing self-oriented goals (e.g., seeking other romantic opportunities) or relational goals (e.g., enhancing relational well-being). This suggests that additional factors may be involved in facilitating promotion oriented individuals to pursue the pro-relationship goals.

Commitment, defined as the intent to persist in the relationship [2], has been shown to orient individuals to forego self-oriented motivations to focus on the broader motivations of the relationship [3]. As such, commitment contributes to a wide variety of pro-relationship maintenance mechanisms such as derogation of alternatives, willingness to sacrifice, forgiveness of a partner, and accommodation (for a review, see [4]). A preliminary correlational study using a small sample by Molden, Finkel, Johnson and Eastwick [5] (see also [6]) has suggested that more romantically committed promotion-focused individuals were less interested in alternative situations to their current relationship, than those who were less committed. In order to offer a systematic empirical test of the theory and to better understand the interplay of self-regulatory processes and relational processes, we conducted one cross-sectional and two experimental studies. We argue that regulatory focus will direct individuals to pursue their goals, with commitment guiding romantically involved individuals to activate their relationship goals and derogate an attractive alternative target. Building upon past research, we used a new direct measure of interpersonal attraction (Studies 1–3), compared our findings to those obtained with a measure of interest in alternative situations used in a previous study (Study 2), and examined whether activating self-oriented goals would inhibit derogation among promotion-focused individuals (Study 3).

To the best of our knowledge, we are the first to revisit the preliminary work by Molden and his colleagues [5,6] by conducting a systematic examination of the phenomenon. For instance, the original findings were based on one study with a small sample size and with self-reported measures of alternatives, whereas we had three studies with larger sample sizes and a measure of initial attraction. We complemented our analyses using two different self-reported measures of interest in alternatives. Unlike the original study, we also experimentally manipulated regulatory focus with two different manipulations, as well as commitment levels. Lastly, we conducted our studies in Portugal in order to examine the phenomenon cross-culturally and take into account possible differences in dating norms and relationship rules (e.g., [7]).

### Relational and personal motives in derogation of alternatives

Broadly, individuals are motivated to fulfill their most basic needs, including those associated with growth, advancement and development, and those associated with security, safety and protection (for a review, see [8]). According to the Regulatory Focus Theory [1], individuals can have two modes of functioning. Individuals can be focused on goal attainment and strive for new opportunities, known as the promotion regulatory focus, in which individuals are oriented to achieve gains (vs. non-gains) and positive outcomes. On the other hand, individuals can be focused on preventing losses and strive for security, known as the prevention regulatory focus, in which individuals are oriented to avoid losses (vs. non-losses) and the occurrence of negative outcomes.

Both types of regulatory focus have been shown to have different influences on interpersonal outcomes. Of interest, regulatory focus seems to differentially impact single and romantically involved individuals on interpersonal attraction. Preliminary evidence presented by Molden and his colleagues [5] (see also [6]) suggest this to be the case. In their studies, the authors measured both types of regulatory focus and computed a difference score, such that individuals could either be predominantly focused on promotion (i.e., being much more promotion oriented compared to prevention) or on prevention (i.e., being much more prevention oriented compared to promotion). In one study, the authors examined single participants in a speed-dating event. Results showed that a predominant promotion, rather than prevention, focus was associated with greater attentiveness to attractive potential dating partners. Because promotion-focused individuals are generally motivated to pursue new opportunities, these opportunities in a speed-dating event reflect the desired goal of finding a potential dating partner. In another study involving both individuals currently in dating relationships and single people, the authors showed that individuals predominantly focused on promotion were more attentive to alternative others compared to those predominantly focused on prevention. Although relationship status did not moderate the results, single participants were asked to recall how they would have answered during their previous romantic relationship, making it uncertain if there are indeed no relationship status differences. Moreover, the sample in this preliminary study included only a small subsample of currently romantically involved individuals (*n* = 34), which could have biased their findings and may not reflect how people in romantic relationships actually behave.

Examining only currently romantically involved individuals in a separate study, however, Molden and colleagues [5,6] showed that commitment interacted with regulatory focus to activate derogation. Participants were asked to evaluate the desirability of alternative scenarios to their current relationship using the item “The alternatives to my current relationship (including being on my own) are desirable”. Results showed that highly committed individuals with a predominant focus on either promotion or prevention were significantly less interested in these alternative scenarios compared to those with low commitment. This negative association was stronger for individuals predominantly focused on prevention. Whereas findings for prevention-focused individuals are in line with the typical pattern of avoiding losses, findings for predominantly promotion focused individuals showing them to be less interested in alternative scenarios is especially relevant because these individuals typically pursue new opportunities [1,8]. Note that in this study, Molden and his colleagues [5,6] did not measure directly interest toward a specific attractive alternative target, but rather interest in alternative scenarios. Still, their findings suggest that goal content matters (see also [9]). For romantically involved individuals, a promotion focus can motivate existing relationship growth and advancement motives when highly committed, presumably making relational goals more salient [3] (see also [10]).

Recent evidence in group processes suggests that promotion can drive individuals to follow group-interests under certain circumstances. For example, Zaal and colleagues [11] have shown that promotion-focused individuals were less likely to pursue self-motivated goals when such goals were aligned with those of other members of the group, than when they were not aligned. No differences were found for prevention focused individuals. Similar behaviors may be expected in the romantic relationship context, with commitment directing promotion individuals to pursue relationship goals, leading to greater derogation of potential alternatives. Furthermore, a predominant focus on promotion has also been to shown to increase the likelihood of having ever been in a relationship, and of currently being in a relationship, compared to a predominant focus on prevention [5]. This suggests that promotion-focused individuals are not only motivated to pursue romantic relationships, but they are also likely to remain in their current relationship and are not necessarily motivated to pursue new romantic opportunities.

In particular, we argue that commitment will be likely to motivate individuals to act in the interest of the relationship, rather than in the pursue of solely self-oriented interests. Our main goal is to test the interaction between regulatory focus and commitment on derogation of an attractive alternative. High levels of commitment should favor the activation of pro-relationship maintenance mechanisms by redirecting the focus of individuals to broader relationship goals [3,12,13]. Therefore, derogation of an attractive person should occur for more committed individuals with a promotion focus. A similar but stronger effect of commitment on derogation should occur for prevention-focused individuals, because they are oriented towards security and avoiding losses.

### Commitment and relational motives

The Investment Model characterizes commitment as having a long-term orientation, intent to persist in a romantic relationship, and forming psychological bonds to the partner [2]. Past research has reliably shown that commitment is crucial in maintaining relationships [14], by driving pro-relationship motives and activating pro-relationship maintenance mechanisms [15]. Commitment has been shown to be effective at transforming self-oriented impulses and interests toward relational goals [3], which in turn facilitates the restoration of relational satisfaction and stability [16].

Research has shown that highly committed individuals are more likely to derogate attractive alternative others, perceiving them as less desirable and reporting less attraction to them, compared to their less committed counterparts [17–19]. Compared to those low on commitment, highly committed individuals are also less attentive to alternative others [20], remember them as being less attractive [21], are better off at suppressing thoughts about them [22], recall fewer positive behaviors from them [23], and engage in less mimicry when interacting with them [24]. Presumably, when individuals are in a relationship, they consider the possibility of engaging in self-motivated behaviors and compare the short-term benefits against the long-term consequences of such behaviors. If a self-motivated behavior is likely to harm the stability of the relationship (e.g., a behavior that leads to infidelity), greater commitment levels serve to transform self-motivations into relational-motivations in order to protect the stability of the relationship [3,25]. For example, sexually unrestricted individuals are less likely to consider engaging in infidelity when highly committed to their relationship [26], and highly committed individuals are less likely to have engaged in sexual infidelity in their current relationship [27,28].

To the extent that individuals are highly committed, their partner and their relationship are also more salient. Research has shown that highly committed individuals spontaneously use more plural nouns, report a greater self-partner overlap, and perceive their relationship to have a more central role in their lives [12]. This greater salience favors the activation of pro-relationship maintenance mechanisms. For instance, Etcheverry and Le [13] asked individuals to complete sentence stems from a commitment scale and measured their reaction times as an indicator of commitment salience. Individuals with shorter reaction times were more likely to remain in their relationship 7 months later, as well as report engaging in accommodation and willingness to sacrifice.

Even though commitment is regarded as a fairly stable construct, Finkel, Rusbult, Kumashiro and Hannon [29] have shown that priming individuals with high or low commitment can induce temporary changes in commitment strength and influence the activation of pro-relationship maintenance mechanisms. In their study, individuals were presented with questions related to their relationship, designed to activate thoughts about their romantic commitment (e.g., high commitment prime: “If your relationship were to end in the near future, what would upset you the most about not being with your partner anymore?”), or presented with questions related to their independent self, designed to activate thoughts regarding to lack of commitment (e.g. low commitment prime: “Describe an activity that you enjoy engaging in when your partner is not around”). Those primed with high commitment were more likely to forgive acts of betrayal from their partner than those primed with low commitment. This is evidence of the casual association between greater commitment and pro-relationship maintenance mechanisms.

In the present article, we argue that commitment operates in conjunction with regulatory focus to activate pro-relationship maintenance mechanisms. In particular, because a focus on promotion activates growth and advancement and because commitment redirects the nature of the goals pursued by romantically involved individuals, we argue that greater levels of commitment will make relational-oriented motives more salient for promotion oriented individuals and activate derogation of an attractive alternative target, whereas lower levels of commitment make self-oriented motives more salient and inhibit derogation. Because individuals in a prevention focus have safety and protection motives and vigilantly try to avoid losses, romantically involved individuals with a prevention focus may be more likely to derogate alternative others, and this should be especially the case for more committed individuals [5,6].

## General overview

The current research sought to conduct a systematic extension of previous findings [5,6] linking the interaction of regulatory focus and commitment on derogation of alternatives (i.e., lower levels of initial attraction towards an attractive target). Specifically, we conducted three studies (one cross-sectional and two experimental) with a larger sample size, experimental induction of regulatory focus and commitment, and new measures of derogation of alternatives, to investigate if romantic commitment will induce promotion oriented individuals to adopt pro-relationship maintenance mechanisms and derogate attractive alternative. A similar association should occur for prevention oriented individuals, with commitment inducing higher levels of derogation of alternatives. However, and based on previous preliminary findings [5,6], this moderation by commitment is expected to be weaker for promotion compared to prevention oriented individuals, given that prevention, with its focus on avoiding losses, is more likely to drive individuals to preserve their existing relationship. We further sought to compare initial attraction to a potential romantic partner between single and romantically involved individuals to assess how promotion (vs. prevention) might direct interest among single people. Finally, we sought to examine this phenomenon cross-culturally by conducting this research in Portugal, which might have different dating norms compared to the USA [7].

Based on our rationale, we expect that:

Hypothesis 1: Regulatory focus interacts with relationship status on initial attraction judgments (Study 1). Specifically,

Hypothesis 1A: Single individuals with a predominant focus on promotion will indicate greater initial attraction to an attractive other, compared to those with a predominant focus on prevention;

Hypothesis 1B: This association between predominant focus on promotion and initial attraction should be weaker, or even disappear, for romantically involved individuals.

For those in a romantic relationship, we also expect that:

Hypothesis 2: Relationship commitment interacts with regulatory focus on initial attraction judgments, such that commitment will activate pro-relationship maintenance mechanisms. Specifically,

Hypothesis 2A: When less committed, promotion-focused individuals should indicate greater levels of initial attraction compared to prevention-focused individuals. This pattern converges with the typical orientation of promotion-focused individuals toward pursuing new opportunities and prevention-focused individuals towards security (Studies 1–3);

Hypothesis 2B: When more committed, promotion- and especially prevention-focused individuals should indicate less initial attraction. Unlike the typical orientation of promotion-focused individuals towards new opportunities, we argue that commitment shifts their typically self-oriented goals into relationship-oriented goals (Studies 1–3).

*Contributions to the literature*. The current research seeks to extend the literature in several important ways. First, we extend past findings by examining the underlying mechanism for how commitment works with promotion focus to redirect individuals in romantic relationships to adopt pro-relationship maintenance mechanisms. Study 1 is the first study to directly compare individuals who are single to those who are in romantic relationships. Preliminary findings [5,6] suggest that individuals predominantly focused on promotion are more interested in potential partners compared to those with prevention focus, presumably to pursue their desired goals. This replicates the typical pattern of promotion-focused individuals as pursuing new opportunities [1]. However, there is a conflict for those in a romantic relationship, because there are self-interests that could conflict with relational interests. In Study 3, we specifically examined whether romantically involved individuals are effective in derogating an attractive alternative (favoring pro-relationship interests), when shared relationship interests are more salient (e.g., common goals for the relationship). In contrast, when purely self-interests are more salient (e.g., goals for the self, regardless of the partner), romantically involved individuals in a promotion focus may not differ from single individuals in their interest in new potential partners. This is an important extension of the literature, because it will reveal how a relationship process (i.e., commitment) can redirect a motivational process (i.e., promotion focus) to maintain the health of a relationship by inhibiting initial attraction.

Second, we also extend the literature by examining this phenomenon more systematically. We used larger sample sizes and a more direct measure of initial attraction, rather than interest in alternative scenarios. We also established a causal direction regarding this phenomenon for the first time by manipulating regulatory focus using two different manipulations (rather than measuring individual differences in regulatory focus strength), and by manipulating for the first time commitment strength in the context of derogation.

Lastly, we examined if promotion focus and commitment interact in a similar way in a different Western culture. This latter novel aspect is relevant given that there might be cultural differences in dating practices between Portugal and the United States, where the preliminary studies were conducted. For instance, although individuals in both countries hold negative attitudes towards extradyadic sex [30,31], infidelity is not uncommon in both countries [27,28,32]. Nevertheless, individuals in the United States have been shown to be more sociosexually unrestricted than in Portugal, meaning that they are more comfortable with, and have more favorable attitudes towards, casual sex [33]. Moreover, there might also be differences in chronic regulatory focus between both countries. Although individuals in Western countries tend to be predominantly promotion-focused, compared to Eastern countries which tend to be predominantly prevention-focused, Higgins, Pierro and Kruglanski [34] showed a greater range in promotion and prevention scores in the West. For instance, individuals in the United States scored higher in promotion than individuals in Italy, who in turn did not differ from individuals in China. This might translate to differences in interpersonal attraction or in the interest in alternative others, especially among promotion-focused individuals.

## Ethics statement

All studies involved human data collection from healthy adult volunteers. The study was reviewed and approved by the CIS-IUL scientific and ethical committee at ISCTE-IUL. There were no physical, financial, social, legal, or other risks connected with the studies. All studies were noninvasive, no false information was provided and results were analyzed anonymously. In all studies participants read the description and purpose of the study, were informed that they could leave the study at any point and could only proceed after providing written informed consent. At the end of each study, all participants were thanked and provided with a short debriefing.

## Study 1

In a cross-sectional study, we examined the association between regulatory focus and initial attraction to an attractive alternative target for individuals who were either single or in a relationship.

### Participants

Participants were Portuguese heterosexual individuals (*N* = 230; 75.2% female; *M*_age_ = 23.25, *SD* = 4.44) who voluntarily took part on an online survey. Nearly half of the participants were single and not in a relationship (*n* = 103; 70.9% female) and the remaining participants were in an exclusive romantic relationship (*n* = 127; 78.7% female). Out of the entire sample, 45.7% was dating and 9.6% was married (*M*_length_ = 53.70 months, *SD* = 77.81). There were no gender differences according to relationship status, χ^2^ (1) = 1.89, *p* = .169.

### Measures

#### Regulatory focus questionnaire

Participants were asked to report how frequently a series of events occur in their lives [35]. Half of the events reflect a prevention focus (five items; e.g., “Not being careful enough has gotten me into trouble at times”) and the other half reflect a promotion focus (six items; e.g., “Compared to most people, are you typically unable to get what you want out of life?”). Responses were given on 7-point scales (e.g., 1 = *Never or seldom*, 7 = *Very often*). The items were translated to Portuguese by the team of researchers and back-translated by a Portuguese and English fluent speaker. Disagreements were resolved through discussion (90% agreement). The measure showed good reliability (*α*_Prevention_ = .78 and *α*_Promotion_ = .73), with a modest positive correlation between both scales, *r*(230) = .16, *p* = .014 (see also [35]).

#### Initial attraction

This measure was developed in Portuguese [36]. Participants were asked to indicate their initial attraction towards the target (five items; *α* = .93; e.g., “I would like to know this person better”). Responses were given on 7-point scales (1 = *Not at all*, 7 = *A lot*).

#### Relationship commitment

This measure was previously validated in Portugal [37]. Romantically involved participants were asked to indicate their degree of commitment to their current relationship (seven items; *α* = .93; e.g., “I want our relationship to last for a very long time”) [14]. Responses were given on 7-point scales (1 = *Do not agree at all*, 7 = *Agree completely*).

### Procedure

Individuals were invited (e.g., via email, social network websites) to participate in an online study. By clicking on the provided hyperlink, individuals were directed to a secure webpage and were then told they would be taking part in two ostensibly independent studies, the first on how people deal with daily events, and the second on how individuals process visual information. The questionnaire started with standard demographic questions (e.g., age, relationship status, sexual orientation), followed by the regulatory focus measure. After this, a black and white headshot (3 x 4 inches) of an opposite sex attractive target with a neutral facial expression (pre-tested; [36]] was presented for 5 seconds, followed by the initial attraction measure. Because commitment refers to a specific romantic relationship, only romantically involved participants were additionally asked to indicate their commitment to their romantic relationship.

### Results

#### Preliminary analyses

We computed a Regulatory Focus Index (RFI) by subtracting prevention scores from promotion scores, such that higher scores reflect a predominant focus on promotion. This scoring method is commonly used in the literature as a standard measure of predominant regulatory focus [5,6,35,38–42].

Descriptive information of measures is provided in Table 1. Results show that RFI was positively correlated with initial attraction measure for single participants, *r*(103) = .21, *p* = .031. For romantically involved individuals, there were no significant associations between RFI and initial attraction, *r*(127) = -.15, *p* = .110, RFI and commitment, *r*(127) = .07, *p* = .434, or initial attraction and commitment, *r*(127) = -.10, *p* = .267.

**Table 1:** Descriptive information for single participants and for romantically involved participants (Study 1).

	Single	Romantically involved		
	*M* (*SD*)	*M* (*SD*)	*t* test	Cohen’s *d*
1. RFI	4.59 (5.34)	5.35 (5.23)	-1.09	-
2. Initial attraction	3.64 (1.22)	2.81 (1.24)	5.12^***^	0.68
3. Commitment	-	6.13 (1.15)	-	-

Degrees of freedom for *t*-statistics = 228.

***p ≤ .001.

#### Regulatory focus and relationship status

To test the moderation by relationship status in the association between regulatory focus and initial attraction (Hypothesis 1) we ran a linear regression model using PROCESS bootstrapping macro for SPSS [43] with 5,000 resamples and 95% bias-corrected standardized bootstrap confidence intervals (CI). RFI score, relationship status (coded 0 = single, +1 = romantically involved) and the interaction term were entered as predictors, with initial attraction as the criterion (Model 1). Variables were centered prior to the analysis.

**Fig 1:**
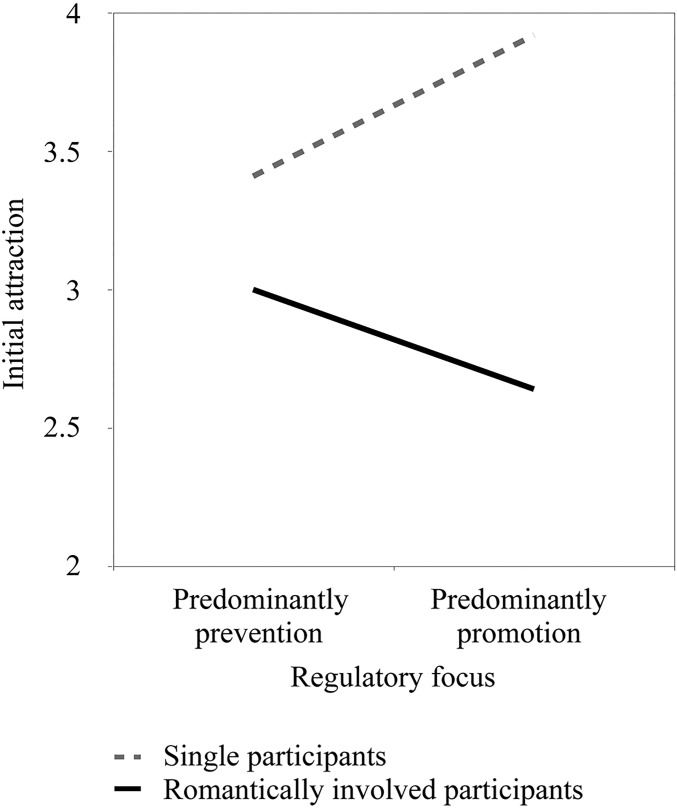
Association between initial attraction and predominant regulatory focus as a function of relationship status (Study 1).

Results showed a significant main effect of relationship status, *b* = -0.85, *SE* = .16, *t*(226) = -5.22, *p* < .001, 95% CI [-1.17, -0.53], such that romantically involved participants reported less initial attraction for the target. There was no main effect of RFI, *p* = .837. More importantly, there was a significant interaction between RFI and relationship status, *b* = -0.08, *SE* = .03, *t*(226) = -2.70, *p* = .008, 95% CI [-0.14, -0.02]. Simple slopes analyses revealed a positive association between a predominantly promotion focus and initial attraction for single participants, *b* = 0.05, *SE* = .02, *t*(226) = 2.16, *p* = .032, 95% CI [0.01, 0.09], whereas for romantically involved participants this association was non-significant, *b* = -0.03, *SE* = .02, *t*(226) = -1.63, *p* = .104, 95% CI [-0.07, 0.01] (see Fig 1). These results remained the same after controlling for gender.

#### Regulatory focus and commitment

To examine if the non-significant association between a predominant promotion focus and initial attraction among romantically involved participants was moderated by commitment (Hypothesis 2), a second linear regression was conducted. As only romantically involved individuals reported their commitment, only these participants were considered for this analysis. RFI and commitment scores, as well as the interaction term were entered as predictors, with initial attraction as the criterion (PROCESS Model 1). Variables were centered prior to the analysis.

**Fig 2:**
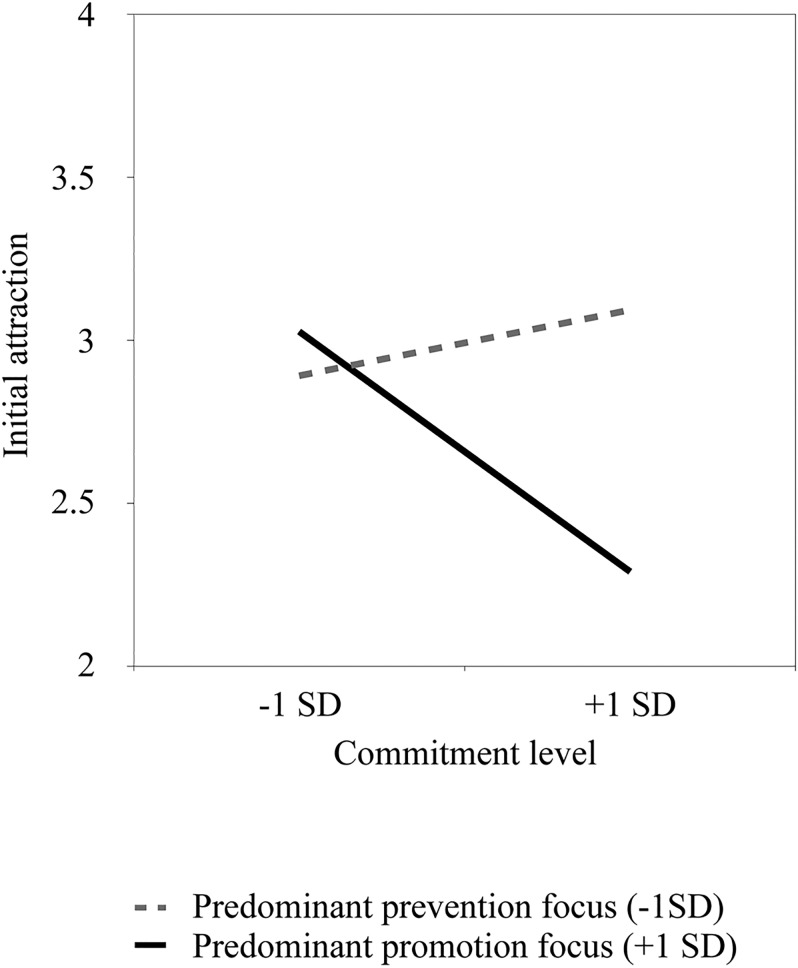
Association between initial attraction and commitment level as a function of predominant regulatory focus (Study 1).

There was no main effect of RFI, *p* = .127, or commitment, *p* = .222. Instead, results showed an interaction between the factors, *b* = -0.04, *SE* = .02, *t*(123) = -2.09, *p* = .039, 95% CI [-0.08, -0.01]. Simple slope analyses revealed that initial attraction only decreased with greater levels of commitment for individuals predominantly focused on promotion (+1 *SD*), *b* = -0.32, *SE* = .14, *t*(123) = -2.25, *p* = .026, 95% CI [-0.60, -0.04], but not for individuals predominantly focused on prevention (-1 *SD*), *t* < 1 (see Fig 2). A floodlight analysis using the Johnson–Neyman technique [44] showed that this moderation was significant only for commitment levels above 6.39, *b* = -0.04, *SE* = .02, *t*(123) = -1.98, *p* = .050, 95% CI [-0.08, 0.00]. Additional analyses with gender and relationship length as covariates yielded a similar pattern of results.

To examine the interaction between each type regulatory focus and commitment in the activation of derogation, we replicated these analyses entering both promotion and prevention scores separately in the same model, alongside commitment (PROCESS Model 2). Simple slope analyses show that increases of commitment were associated with a significant decrease in initial attraction for individuals predominantly more focused on promotion (+1 *SD*) and less focused on prevention (-1 *SD*), *b* = -0.43, *SE* = .188, *t*(121) = -2.26, *p* = .025, 95% CI [-0.80, -0.05]. No other results reached significance. These results show that derogation was driven by an interaction between commitment and a greater focus on promotion, rather than prevention. We opted to use the composite RFI score because: a) it provides a clearer pattern for our results; b) results from Study 1 are more easily compared with those from Studies 2 and 3, in which we induce promotion and prevention; and c) this score has been extensively used in the literature [5,6,35,38–42].

### Discussion

Single individuals reported greater initial attraction when predominately focused on promotion, but not on prevention. For romantically involved individuals, there was no association between regulatory focus and initial attraction, but rather, commitment moderated the association between regulatory focus and initial attraction. Individuals predominantly focused on promotion showed greater derogation of the attractive alternative when highly committed to their relationship compared to those with low commitment. Unlike the preliminary findings of Molden and his colleagues [5], no differences were found for individuals predominantly focused on prevention. However, as the authors discussed, a general measure of perceived alternative scenarios was used, which does not necessarily reflect romantic interest, whereas in our study we asked participants to report their initial attraction to a specific attractive target.

Other possible sources of concern include gender differences or translation issues associated with the regulatory focus measure. However, gender was not a significant covariate in either of our analyses, and the regulatory focus measure went through the typical process of translation before it was used. It is also possible that differences in the results might be accounted by cultural differences in dating practices. For instance, cross-cultural studies have established that individuals in the United States have more unrestricted attitudes towards casual sex than those in Portugal [33]. As such, it is possible that in the preliminary study using only romantically involved participants [5,6], those predominantly prevention-focused interpreted “alternative to my relationship” as potential alternative sexual partners, which activated greater derogation due to a greater perceived threat to the relationship. In contrast, our measure of initial attraction does not contain items associated with sexuality, and reflects interest in knowing more about another person [36,45]. This may be the reason for the lack of association between commitment and initial attraction among these individuals. Because we did not use the same measure as Molden and colleagues [5,6], results are not comparable.

In Study 2 we focused only on romantically involved individuals, and examined in greater detail the association between regulatory focus and derogation of alternatives. We experimentally manipulated regulatory focus for the first time in this context, to gather evidence of causality between regulatory focus and derogation. We added two measures of perceived quality of alternatives to be able to compare our findings to past findings [5,6] and to examine results for prevention-focused individuals in greater detail.

## Study 2

In this study, we experimentally induced individuals in a promotion or a prevention focus by asking them to write a short essay about their hopes and aspirations, or about their duties and obligations, respectively. This is a non-intrusive and reliable methodology extensively used in the literature [46–48]. Following this manipulation, we asked individuals to report their initial attraction to an attractive target and their romantic commitment.

### Method

#### Participants and design

Participants were 55 heterosexual individuals (78.2% female; *M*_age_ = 20.18, *SD* = 3.85) who were in an exclusive romantic relationship (92.7% dating and 7.3% married; *M*_length_ = 22.27 months, *SD* = 24.49).

Individuals voluntarily participated in exchange for course credits and were randomly assigned to conditions in a 2 Regulatory focus (prevention vs. promotion) between-subjects factorial design.

#### Measures

All measures were the same as in Study 1 with two exceptions. Measures of quality of alternatives and attentiveness to alternative others were added.

#### Quality of alternatives

This measure was previously validated in Portugal [37]. Participants were asked to indicate the quality of alternative scenarios to their current relationship (five items; *α* = .90; e.g., “My alternatives are attractive to me [dating another, spending time with friends or on my own, etc.]”) [14]. Responses were given on 7-point scales (1 = *Do not agree at all*, 7 = *Agree completely*).

#### Attentiveness to alternatives

Participants were asked to which extent they spend time attending to potential alternative others in general (six items; e.g., “I am distracted by other people that I find attractive”) [20]. Responses were given on 7-point scales (1 = *Rarely*, 7 = *Frequently*). The items were translated to Portuguese by the team of researchers and back-translated by another speaker who is fluent in both Portuguese and English. Disagreements were resolved through discussion (95% agreement). The measure showed good reliability (*α* = .88).

#### Procedure

Upon arrival at the lab, participants were told they were taking part in two ostensibly independent studies. The first study was presented as a pre-testing of material to use in future research and the second study was ostensibly to focus on how individuals process daily visual information.

Regulatory focus was manipulated by asking participants to write a short essay. In the prevention focus condition, they were asked to write about their current duties and obligations. In the promotion focus condition they were asked to write about their hopes and aspirations [46]. Upon completing the first task (regulatory focus induction), participants were redirected to an online questionnaire. Procedure and materials were similar to Study 1. Briefly, participants indicated their attraction to the attractive target, indicated their relationship commitment and were presented with the regulatory focus measure (which served as manipulation check). Finally, participants were presented with the measures of quality of and attentiveness to alternatives.

### Results

#### Preliminary analyses

Means, standard deviations and correlations between the measures are provided in Table 2. Results show that initial attraction was only positively correlated with attentiveness to alternatives, *p* = .017. Results also showed that commitment was negatively correlated with quality of alternatives and attentiveness to alternatives, both *p* < .001. Both measures of alternatives were positively correlated, *p* < .001.

**Table 2:** Descriptive information (Study 2).

		Correlations		
	*M* (*SD*)	1	2	3
1. Initial attraction	3.35 (0.90)	-		
2. Commitment	5.05 (1.64)	-.12	-	
3. Quality of alternatives	3.30 (1.56)	.20	-.59^***^	-
4. Attentiveness to alternatives	3.09 (1.47)	.32^*^	-.63^***^	.70^***^

* *p* ≤ .050.

*** *p* ≤ .001.

#### Regulatory focus and initial attraction

To examine if commitment level moderates the effect of regulatory focus on initial attraction (Hypothesis 2), a linear regression was conducted using PROCESS. Regulatory focus manipulation (coded 0: prevention, +1: promotion), commitment scores and the respective interaction were entered as predictors, with initial attraction as the criterion (Model 1). Variables were centered prior to the analysis.

**Fig 3:**
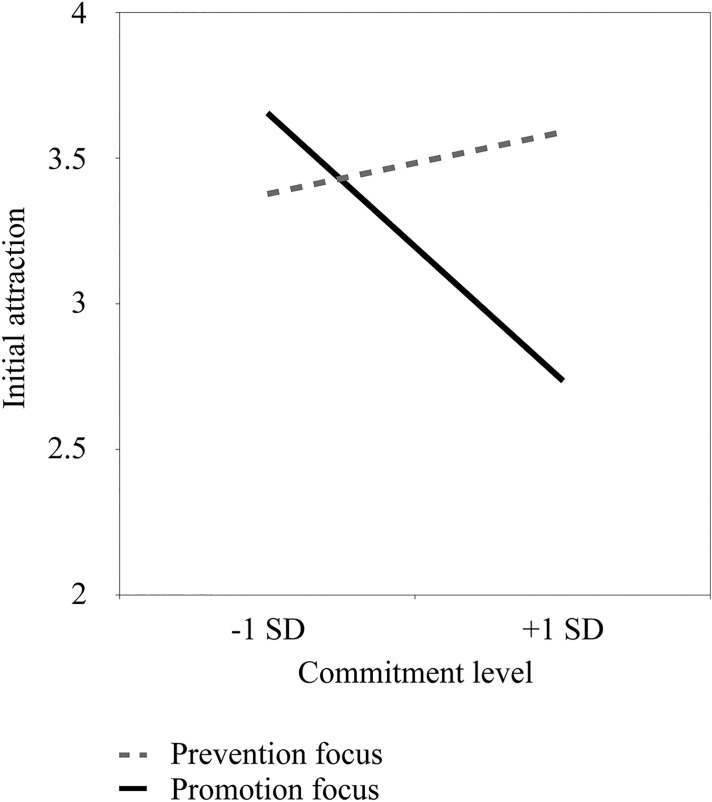
Association between initial attraction and commitment level as a function of the regulatory focus manipulation (Study 2).

Results showed no main effect of regulatory focus, *p* = .223, or commitment, *p* = .138. The expected interaction between these factors was significant, *b* = -0.34, *SE* = .15, *t*(51) = -2.36, *p* = .022, 95% CI [-0.64, -0.05]. Simple slope analyses showed that initial attraction decreased for individuals induced in a promotion focus with greater levels of commitment, *b* = -0.28, *SE* = .11, *t*(51) = -2.47, *p* = .017, 95% CI [-0.51, -0.05]. This effect was not significant for those induced in a prevention focus, *t* < 1 (see Fig 3). A floodlight analysis [44] showed that this moderation was significant only for commitment levels above 5.87, *b* = -0.51, *SE* = .25, *t*(51) = -2.01, *p* = .050, 95% CI [-1.01, 0.00]. An additional analysis with gender and relationship length as co-variates yielded the same pattern of results.

#### Regulatory focus and alternatives

We conducted two additional linear regressions to examine the association between regulatory focus and the perception of alternatives. In the first we entered quality of alternatives as the dependent variable (PROCESS Model 1). Results showed a main effect of commitment, *b* = -0.58, *SE* = .11, *t*(51) = -5.33, *p* < .001, 95% CI [-0.80, -0.36], such that highly committed individuals indicated low quality of alternatives. Neither regulatory focus, *p* = .716, nor the interaction between the factors, *p* = .288, reached significance.

In the second linear regression, we entered attentiveness to alternatives as the criterion (PROCESS Model 1). Again, there was only a main effect of commitment, *b* = -0.60, *SE* = .10, *t*(51) = -6.26, *p* < .001, 95% CI [-0.79, -0.41], such that highly committed individuals indicated being less attentive to alternative others. No significant effects emerged from regulatory focus, *p* = .324, or from the interaction between the variables, *p* = .124. Additional analyses with gender and relationship length as covariates yielded the same pattern of results for both dependent measures.

### Discussion

We replicated our previous findings regarding the association between regulatory focus and attraction. By manipulating regulatory focus, we showed a negative association between promotion and initial attraction, but only among more committed individuals. Again, the association was non-significant for prevention-focused individuals.

We also examined whether this pattern of results could be accounted by differences in the dependent measures. Our results showed that more committed individuals, either induced into a prevention or into a promotion focus, were less interested in alternatives and less attentive to others in general, than their less committed counterparts. It is possible that the lack of interaction between regulatory focus and commitment levels in these measures was partly due to the order in which our dependent measures were presented to participants. Indeed, both evaluations of alternatives were presented last, after assessment of regulatory focus, and participants may have had their original levels of regulatory focus restored. If so, and because participants were on average in a relationship for almost two years (*M*_lenght_ = 22.27 months), commitment may have had a similar impact in initial attraction for individuals induced into a promotion- and prevention-focus, and replicated the typical pattern of results found in the literature [17–20]. It is also possible that whereas both measures assess an overlapping construct, initial attraction assesses a slightly diferent construct. The general pattern of correlations between the measures supports this notion (see Table 2).

In this study, we assessed commitment after the initial attraction judgments. This may be a weakness in our design, since it could be argued that being faced with an attractive target influenced commitment levels. Even though commitment levels are typically regarded as being fairly stable [29], we addressed this issue in the next study by temporarily manipulating commitment strength before the presentation of the attractive target. Specifically, we examined whether inducing individuals in a promotion or in a prevention focus and priming them with high or low commitment also prompts derogation of an attractive target.

## Study 3

In this study, we temporarily induced regulatory focus by presenting individuals with another task that has been shown to be effective at inducing promotion and prevention (i.e., solve a paper-and-pencil maze) [49]. We also manipulated commitment by asking individuals to focus on different goals. This is a non-intrusive and reliable methodology originally presented by Finkel and colleagues [29], designed to activate thoughts about interdependence and commitment or to activate thoughts about dependence and lack of commitment, respectively.

This procedure allows us to directly examine our hypothesis that romantically involved individuals who are more committed redirect their motivations to pursue relational goals, instead of self-oriented ones. To test this, we temporarily activated relational goals (high commitment prime) or self-oriented goals (low commitment prime) and examined their influence in derogation.

### Method

#### Participants and design

A sample of heterosexual individuals (*N* = 132; 82.6% female; *M*_age_ = 21.67, *SD* = 4.20) who were involved in a romantic relationship (95.5% dating and 4.5% married; *M*_length_ = 35.05 months, *SD* = 39.60) voluntarily took part in this study in exchange for course credits. They were randomly assigned to conditions in a 2 Regulatory focus (promotion vs. prevention) x 2 Commitment prime (low vs. high) between-subjects factorial design.

#### Procedure and measures

The procedure was similar to Study 2, except for the manipulations of regulatory focus and commitment. The regulatory focus manipulation was presented as a pre-test of material to be used in future research and participants were asked to solve a paper-and-pencil maze. In the prevention focus condition individuals were asked to complete a maze designed to move them towards avoiding a negative consequence (guiding a mouse to escape the maze avoiding an owl). In the promotion focus condition they were asked to complete a similar maze but intended to move them towards a desire end state of goal attainment (connecting a mouse with a piece of cheese) [49]. Following this task, commitment manipulation was introduced and participants were asked to list their goals and aspirations. Half of the participants were asked to list five goals and aspirations they have only for themselves (low commitment condition) and the other half was asked to list five goals and aspirations they have in common with their partner (high commitment condition) [29]. The remaining measures and materials were the same as Study 1. Briefly, participants indicated their attraction to the attractive target and this was followed by the same measures of commitment and regulatory focus. These measures served as manipulation checks. At the end, participants were thanked and provided with a debriefing.

### Results

#### Manipulations checks

Results of a 2 Regulatory focus (promotion vs. prevention) x 2 Commitment prime (low vs. high) MANOVA showed the success of our manipulations. There was a main effect of the regulatory focus manipulation on RFI scores, *F*(1, 127) = 4.71, *MSE* = 119.85, *p* = .032, *η*_p_^2^ = .04, but not on commitment scores, *F*(1, 127) = 1.72, *MSE* = 1.83, *p* = .192, *η*_p_^2^ = .01. Participants induced in a promotion focus (cheese maze) reported being significantly more promotion-focused (*M* = 5.77, *SD* = 4.88) than participants induced in a prevention focus (owl maze) (*M* = 3.87, *SD* = 5.14).

Results also showed a main effect of commitment prime on commitment scores, *F*(1, 127) = 5.08, *MSE* = 5.38, *p* = .026, *η*_p_^2^ = .04, but not on RFI scores, *F* < 1. Participants primed with high commitment reported being significantly more committed (*M* = 6.39, *SD* = 0.92) than participants primed with low commitment (*M* = 5.98, *SD* = 1.12). The interaction between the factors was not significant on both measures, both *F* < 1.

#### Regulatory focus and derogation

**Fig 4:**
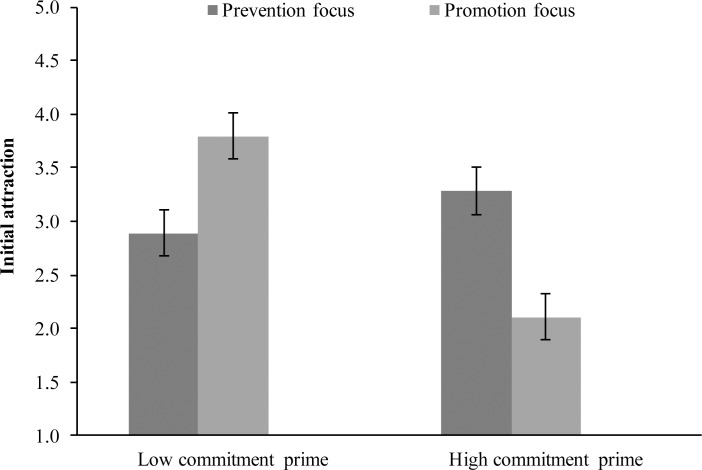
Initial attraction as a function of commitment and regulatory focus manipulations (Study 3).

To examine Hypothesis 2, a 2 Regulatory focus (prevention vs. promotion) x 2 Commitment prime (low vs. high) ANOVA was conducted on initial attraction. Findings showed a significant main effect of commitment prime, *F*(1,128) = 8.91, *MSE* = 13.76, *p* = .003, *η*^2^_*p*_ = .07, but not of regulatory focus, *F* < 1. More importantly, a significant interaction between the factors emerged, *F*(1,125) = 23.01, *MSE* = 35.53, *p* < .001, *η*^2^_*p*_ = .16 (see Fig 4).

Planned contrasts show that participants induced in a promotion focus reported less initial attraction when primed with high commitment (*M* = 2.11, *SD* = 0.92) than when primed with low commitment (*M* = 3.79, *SD* = 1.39), *t*(128) = -5.51, *p* < .001, *d* = 0.97. No differences for initial attraction emerged for participants induced in a prevention focus when primed with high (*M* = 3.28, *SD* = 1.45) or low commitment (*M* = 2.89, *SD* = 1.16), *t*(128) = 1.28, *p* = .203. An additional analysis with gender and relationship status as covariates yielded the same pattern of results.

### Discussion

Results from this study supported our main hypothesis (Hypothesis 2) and also extended results from previous studies. First, the non-significant association between regulatory focus and initial attraction was experimentally replicated with a different manipulation of regulatory focus, suggesting the robustness of the effect. Second, in contrast with our former two studies, we found a main effect of commitment on initial attraction. It is possible that this inconsistency is a reflection of the manipulation of commitment, rather than the measurement of natural levels of commitment. Third, replicating our previous results, there were no differences in derogation for individuals induced in prevention, regardless of commitment prime. Lastly, participants induced in promotion and primed with high commitment showed derogation. Listing relational goals and aspirations may have resulted in the desire for relationship growth, thus prompting derogation of an attractive alternative target. When primed with low commitment, promotion-focused individuals did not seem to activate derogation and instead reported greater initial attraction. By being asked to list self-oriented goals and aspirations, this manipulation may have resulted in making participants desire the pursuit of an attractive alternative. This finding then parallels the results of single individuals found in Study 1.

## General discussion

In the present research, we extended the literature by examining the association between regulatory focus, commitment and initial attraction to an attractive target, and by providing causal evidence for this association. Specifically, we showed that commitment interacts with promotion to activate this pro-relationship maintenance behavior. Not only have we extended the available preliminary evidence to include a more specific measure of initial attraction, rather than evaluation of alternative scenarios, we have also examined these effects cross-culturally.

The cross-sectional findings presented in Study 1 showed that whereas single individuals predominately focused on promotion experienced greater initial attraction to an attractive target, romantically involved individuals who are predominantly focused on promotion experienced less initial attraction, but only when more committed to their relationship (Hypothesis 1). Building upon this, Study 2 experimental findings showed that individuals induced in a promotion focus derogated the target and experienced less initial attraction, but only when more, and not less, committed to their relationship. Moreover, both promotion- and prevention-focused individuals indicated less interest in alternative situations and less attentiveness to others when more, but not less, committed.

This is an important extension of the literature, as these lines of research are typically studied separately and do not examine the interaction between commitment and regulatory focus. An exception is the research by Molden and colleagues [5] (see also [6]), showing a link between regulatory focus and interest in alternatives. For romantically involved individuals, the authors have shown a link between a predominant focus on promotion and interest in broader alternative situations. On our side, we extended these findings by specifically presenting an attractive person and showing that single people predominately focused on promotion were more attracted. For those in a relationship, no positive association between a predominant focus on promotion and initial attraction. However, this link was moderated by commitment, such that more committed individuals reported less attraction than their less committed counterparts.

More importantly, Study 3 further showed that whereas individuals induced in a promotion focus engaged in greater derogation when primed with high commitment compared to those primed with low commitment. These findings provide support for our argument that commitment influences which type of goals are activated–self or relational–and that the motive to pursue either set of goals have different impacts on the activation of pro-relationship maintenance mechanisms (Hypothesis 2). Indeed, promotion-focused individuals for whom relational goals were more salient derogated the attractive target. In contrast, promotion-focused individuals for whom self-goals were more salient showed a similar pattern as the one found for single individuals, that is, greater initial attraction for the attractive target. This also extended the literature by showing that, in contrast to single individuals, promotion motives for romantically involved individuals may become related to relational growth and advancement, with commitment serving as a driving force. Commitment helps individuals to transform self-oriented motives and to consider broader relational motives, such as through accommodating poor partner behaviors [3]. Highly committed individuals also focused on promotion may consider that their goals for growth and advancement are in tandem with relational growth [50]. Thus, these individuals may be especially aware of a potential harm to the stability of their relationship when confronted with an attractive person, and thus engage in greater derogation.

Past studies have shown a link between commitment salience and accommodation, sacrifice and forgiveness [13,29]. Presumably, more committed individuals place a greater emphasis on their commitment, and their relationship assumes a more central role in their lives [12], compared to less committed individuals. We extended this to derogation and showed that commitment salience works with a promotion focus to activate derogation, but most notably that commitment salience emerges as a boundary condition for the activation of this pro-relationship maintenance mechanism when in a promotion focus. This is a highly relevant aspect for romantic relationships literature, as infidelity is one of the most prevalent causes for break-up [51]. To the extent that individuals are more committed to their relationship, they are more focused on developing their relationship well-being [3], their partner is more salient in their daily lives [12], and they are more likely to guard against potential threats that can jeopardize the stability of their relationship [14].

This converges with findings using a modified version of the regulatory focus construct, proposed by Winterheld and Simpson [52]. The authors showed that assessing (or inducing) romantic relationship promotion or prevention orientations (e.g., “I often think about how I can achieve [or create] a successful relationship”) influenced how individuals perceived conflicts, and which resolution strategies they adopted. For instance, the authors showed that, in contrast to relationship prevention-focused individuals, those with a relationship promotion orientation perceived their partners as more supportive and approached conflicts with more creative resolution strategies. As it was the case in our studies, these findings support the notion that relationship promotion-focused individuals are motivated by relationship interests. Also, Zou, Scholer and Higgins [53] have shown that promotion-focused individuals who had a larger gain after an initial investment were more likely to take a conservative approach in the next investment than those who had a smaller gain, in order to maintain their gains. Extending these findings to romantic relationships, more committed individuals may perceive they have progressed towards achieving a stable and committed relationship, thereby adopting a strategy to prioritize and maintaining its stability. When faced with a potential new romantic opportunity, more committed promotion-focused individuals seem to transform self-oriented motivations to relational-oriented motivations, and are therefore guided by relationship interests.

Individuals in a prevention focus, either predominant or induced, indicated being less interested and attentive to alternatives in general (Study 2). Using a more specific measure, however, no association was found between prevention focus and initial attraction when considering the attractive target (Studies 1–3). This suggests that highly committed individuals with this regulatory focus are not likely to use derogation when faced with a specific alternative person (at least when presented in a photo). They seem likely, however, to use derogation when thinking about alternative scenarios and their typical behavior when encountering potential alternative others in their daily lives (similar to promotion-focused individuals). This might be related to the fact that the initial attraction measure assesses a construct related to liking to an unknown target, whereas the other measures assesses interest in specific alternative scenarios or targets already encountered. For instance, it is possible that only recalling a personal experience of encountering an attractive other is perceived as a threat, with commitment subsequently activating derogation. Future research should seek to examine prevention oriented individuals to a greater extent, using other measures of interpersonal attraction (e.g., physical attraction, sexual desire).

It is also possible that a focus on prevention is related to other pro-relationship maintenance mechanisms. For instance, forgiveness is oriented to protect the relationship from an actual threat posed by the partner that has implications for harming the relationship. Hence, commitment to the relationship should be especially relevant for prevention-focused individuals to deal with such situation and avoid further losses made salient by partner transgression. Indeed, Molden and Finkel [54] have shown that the activation of forgiveness is moderated by commitment among prevention-focused individuals and moderated by their trust in the partner among promotion-focused individuals. Derogation, however, is oriented to protect the relationship from a potential external threat that occurs without partner involvement. For instance, it is possible that prevention-focused individuals with greater self-trust about (the lack of) extradyadic interests derogate an attractive alternative more easily than those who are less trusting about such interests. Future research should seek to further examine differences in these pro-relationship maintenance mechanisms.

Our research contributes more broadly to understanding the role of regulatory focus in relationships [9]. Despite the influence that a promotion focus has on thriving and seeking for new opportunities [1], this should be examined by considering the broader social context. In the context of romantic relationships, greater commitment transforms personal into relational motives and redirects the focus to promote relational growth. Such promotion is incompatible with the interest and pursue of an alternative partner or new romantic opportunities. In the context of friendships, in contrast, it is possible that greater commitment to a close friend may not be associated with derogation of a potential new close friend, in the sense that two close friends are not necessarily incompatible. Future research could focus on this possibility.

Our research can also have implications for practice in couple therapy. Highly committed promotion-focused individuals are more likely to protect their relationship against potential external threats. This should be especially the case when commitment is more salient. Therefore, professionals can draw on these findings to further develop existing intervention programs focused on individual differences in regulatory focus and on strategies that foster relational promotion. As our research suggests, it is important not only to create strategies to help individuals focus on their commitment when faced with a potential alternative target (e.g., having individuals think about their partner), but also work on creating strategies to induce individuals into a promotion focus in such situations.

### Limitations and future research

Despite these contributions, our research is not without limitations. One limitation of our paradigm is the artificiality of the attraction target, shown only by one photograph of the face. Participants were not expecting to meet the target, so there may not have been as much of a threat to their current relationship. Moreover, some people may genuinely not have found the target to be attractive. Therefore, future research should seek to examine more realistic situations (e.g., having multiple targets in a social networking site) and include measures of actual pro-relationship maintenance behaviors (e.g., making sacrifices; spending quality time together). Moreover, most participants in our studies were women and conclusions regarding possible gender differences, or lack of thereof, should be taken with caution. Even though gender was not a significant co-variate in either of our studies, and no gender differences were also reported in the preliminary studies [5,6], future research should seek to have a more balanced distribution of men and women across studies.

Another limitation has to do with the manipulation of commitment levels. This manipulation had already been used by other researchers with effective results [29], but recent replication efforts have suggested that there may be flaws or that it is not always reproducible [55] (but see [56]). In our study, asking individuals to list goals and aspirations may have created a potential confound with promotion-focus. However, this is unlikely as our manipulation of regulatory focus was not explicitly related to goals and aspirations and findings of a multivariate analysis showed that the manipulations of regulatory focus and commitment were orthogonal. Nevertheless, future studies should seek to develop alternative manipulations of commitment levels.

Another potential limitation has to do with the stages of a relationship. Couples who are in early stages of a relationship may differ from couples who have greater investments in their relationships, or who are married or cohabiting. Due to sample restrictions, we used relationship length as a proxy of relationship stage, but this variable did not emerge as a significant covariate. Nonetheless, future research should seek to examine to a greater extent possible differences across relationship stages. To generalize to a wider population, future research should also be conducted in different cultures and take into account individuals differences in sociosexuality levels along with other factors such as attitudes toward infidelity. Particularly, findings of this research may need to be replicated in the United States.

Finally, another potential limitation concerns not taking into account partner goal support. Our results from Study 3, where individuals primed with low commitment were asked to focus on their personal goals and aims, revealed that such prime led individuals to be less likely to derogate the attractive alternative other. Extending these findings to the literature on goal support, the pursuit of self-oriented goals by romantically involved individuals can often lead to negative relational consequences [57], for instance meeting potential alternative partners or causing partners to share less similarities. However, support for self-oriented goals increases relationship well-being in unmarried couples [58]. Therefore, having partner support may make people more committed to their relationship and focus on relational goals even in such situations. This could even be translated to the interpersonal attraction context, if both partners have previously discussed and agreed upon non-monogamous norms. In this case, pursuing such self-oriented motives would not be incompatible with promoting the relationship well-being (for instance, see [28]). Future research could seek to expand on this.

## Conclusion

Following recent recommendations and guidance about the importance and need for having high quality replications [59], our set of studies combined different innovative methodologies to strengthen both internal and external validity of a past preliminary research findings and to extend past findings [5,6]. Using cross-sectional (Study 1) and experimental designs (Studies 2 and 3), our results add to a growing body of research focused on the importance of regulatory focus to the development and maintenance of interpersonal relationships [9], and more specifically how this focus can impact the activation of pro-relationship maintenance mechanisms such as forgiveness [54]. Specifically, whereas single individuals felt greater initial attraction to an attractive target when more promotion-focused, commitment decreased initial attraction among romantically-involved individuals with a promotion focus, presumably because they favor relationship goals and are less open to alternative partners. Their less committed counterparts, in contrast, felt more initial attraction, presumably because they favor self-goals, similar to single individuals.

Unlike forgiveness that seems to be activated to prevent the loss of the relationship following a transgressive act from the partner, derogation seems to be activated to promote the growth of the relationship and guard against an external threat posed to oneself. The fact that individuals are able to guard against this perceived threat, based on their level of commitment, can arguably create a sense of security and trust in oneself, and set the tone for future similar situations and prevent infidelity [25]. Developing further research on the interaction between commitment and regulatory focus, and extend it to other pro-relationship maintenance mechanisms, will allow researchers and professionals to deepen their understanding on the dynamics underlying romantic relationship and the motives that hold relationships together.

The formation and maintenance of romantic relationships have always been surrounded by a certain mystery. Similar to the effort individuals have to put in their relationships for them to thrive, researchers are constantly making new efforts to understand why some individuals endure in their relationships, while others decide to go separate ways with their partner. With this research, we contributed to a more comprehensive understanding of this phenomenon, unveiling the role of regulatory focus and commitment in the derogation of attractive targets.
